# Mental health and BMI in children and adolescents during one year in obesity treatment

**DOI:** 10.1186/s12887-024-04835-7

**Published:** 2024-06-26

**Authors:** Katrine Decker Iversen, Trine Pagh Pedersen, Mette Rasmussen, Maj-Britt Lundsgaard Hansen, Birgitte Højgaard Roikjer, Grete Teilmann

**Affiliations:** 1 Department of Pediatrics, The Children’s Obesity Clinic, Copenhagen University Hospital – North Zealand, Hillerød, Denmark; 2 National Institute of Public Health, University of Southern Denmark, Copenhagen, Denmark

**Keywords:** Childhood, Adolescents, Obesity, Mental health

## Abstract

**Background:**

Mental health plays a major role in children and adolescents with obesity. The aim of this study was (1) to compare mental health in children with obesity with the background population and (2) to investigate if mental health changed during one year in an obesity treatment program.

**Methods:**

Data on self-reported mental health was collected in 107 children and adolescents (mean age 13.2 years) with obesity at first visit in an obesity treatment program and at one year follow-up (*n* = 47). Mental health was assessed by eight questions from the Danish Health Behaviour in School-aged Children (HBSC) questionnaire: (1) self-rated health (2) life satisfaction (3) feeling low (4) body-image (5) loneliness (6) self-esteem (7) self-efficacy and (8) social competence. Data was compared to a reference population based on HBSC data. BMI-SDS was based on Danish reference values.

**Results:**

Children and adolescents with obesity had significantly higher odds of reporting negative body image and feeling low and lower odds of reporting high self-rated health and high self-esteem compared to a reference population. There was no difference between the groups regarding life-satisfaction, social competence, self-efficacy or feeling lonely. There were no significant changes in mental health from first visit to one-year follow-up.

**Conclusion:**

Our findings highlight the mental health difficulties in children and adolescents with obesity, and the importance of addressing these issues in obesity treatment. The results also indicate that children with obesity have psychosocial resources that should be exploited in treatment protocols.

**Supplementary Information:**

The online version contains supplementary material available at 10.1186/s12887-024-04835-7.

## Introduction

Childhood obesity is a growing problem, and we are dealing with a global obesity epidemic [1, 2]. Negative physical health consequences of obesity in childhood and adolescence are well-documented [3–5] and there is strong evidence that childhood obesity tracks into adulthood [6] and is associated with adult morbidity [7]. A major concern is the negative effects of childhood obesity on mental health and wellbeing. It has been reported that children with overweight and obesity are more likely to experience the burden of emotional and behavioural disorders [8–10].

Studies have found that overweight and obesity among children and adolescents is associated with low self-esteem [9, 11], poor peer relationship and increased body dissatisfaction compared to non-obese children and adolescents [10, 11]. Furthermore, childhood and adolescent obesity has been linked to poor self-rated health [12] and lower self-efficacy [13]. Obesity does not only have an immediate effect on children’s wellbeing, but it also effects mental health in a long-term setting [14, 15]. However, the causality between childhood obesity and mental health remains unclear [16, 17].

It is well known that treating obesity among children and adolescents has proven to be difficult. The current golden standard in treatment of childhood obesity is a chronic care treatment model that is delivered in an age-appropriate way and includes culturally sensitive, family-centred lifestyle modifications (diet, physical activity and sedentary behaviour) [18]. However, there is still a high demand for improving the treatment to achieve a significant and clinically meaningful weight development in children and adolescents.

Existing studies on mental health in children and adolescents with obesity typically investigate only few parameters of mental health providing little information on mental health in a more general sense in children and adolescents with obesity. Recent guidelines from American Academy of Pediatrics regarding evaluation and treatment of children and adolescents with obesity concluded that more studies on treatments’ impact on mental health are needed [19].

The purposes of this study was primarily to investigate differences in mental health in children and adolescents with obesity compared to a Danish reference population. Secondly, the study aimed to investigated if different parameters of mental health changed after one year treatment in a chronic-care treatment program for children with obesity.

## Methods

### Design and setting

This is an observational study, conducted in a Danish hospital-based outpatient clinic for children and adolescents with obesity, *The Children’s Obesity Clinic, Department of Paediatrics, North Zealand Hospital, Hillerød*. Inclusion criteria for the mental health study were age between 11 and 17 years old or enrolled in 5th grade, and a BMI above the 99th percentile for sex and age. Participants in this study were recruited from August 2014- December 2016.

### The children’s obesity clinic’s treatment protocol

The clinical examination was based on The Children’s Obesity Clinic’s Treatment protocol, described in detail elsewhere [20]. In brief, the protocol is an individual family-based obesity intervention aiming to improve different aspects in the child’s daily life concerning childhood obesity and prevent future development of obesity.

At the clinical consultation at first visit and at follow-up, height, weight, body fat, muscle mass, waist- and hip circumference as well as blood pressure were measured. Height was measured by Seca 216 stadiometer, to the nearest millimetre, calibrated monthly by use of standard 100 cm measure. Weight was measured with standard calibrated TANITA BC-418 MA to the nearest 0.1 kg without shoes and in light indoor clothing, without the need for calibration before 300.000 measurements [21]. BMI was converted into a standard deviation score (BMI-SDS) using the LMS method based on Danish reference values [22].

The clinical encounter contained an in-depth medical history based on a standardized questionnaire. Information on socioeconomic status (SES), ethnicity and family structure were also reported. SES was categorized according to the National Statistics Socioeconomic Classification into 1–5 groups according to occupation [23]. SES groups were dichotomized into high [1–3] or low (4–5). Family structure was classified by whom the child was living with as follows; (1) living with both parents, (2) disrupted (single or divorced) or (3) alternative structure (foster home, living with other family members etc.). Family structure was dichotomized; (1) living with both parents [1] or (2) disrupted family structure (2–3).

### Assessment of mental health

Mental health was evaluated using the Danish version of the Health Behaviour in School-aged Children Questionnaire (HBSC) a World Health Organisation collaborate study, repeated every fourth year, examining health, wellbeing, social environments and health behaviour in school-aged children [24]. The questionnaire was slightly revised, as questions about sexual- and alcohol habits were left out. If the child experienced any difficulties with understanding or reading questions a research assistant assisted.

We selected eight variables from the HBSC questionnaire to evaluate mental health: (1) self-rated health (2) life satisfaction (3) feeling low (4) body-image (5) loneliness (6) self-esteem (7) self-efficacy and (8) social competence. Table 1. shows each parameter, with the associated question(s), response options and subsequent re-categorizing.

**Table 1:** Mental health domains with the associated question(s), response options and subsequent re-categorizing

Domain	Question	Response options	Re-categorization
**Self-rated health**	“Would you say your health is…?”	“Excellent”, “Good”, “Fair” or “Poor”	“Excellent” => high self-rated health Other = > middle/low
**Life satisfaction**	“Rate your life satisfaction using a 11-step visual analogue scale ranging from worst- to the best possible life (0–10)”	Visual analogue scale ranging 0–10	≥ 9 = > high life-satisfaction ≤ 8 = > middle/low
**Feeling low**	“How often did you experience feeling low in the last six months?”	“About every day”, “Least once a week”, “Almost every week” or “Almost every month”	“About every day” or “Feeling low least once a week” => often feeling low. Feeling low less than every week = > less than every week
**Body-image**	“How do you perceive your body?”	“Too thin”, “Thin”, “Appropriate”, Fat”, “Too fat”	“Fat” or “Too fat” => negative body image Other = > normal or positive
**Loneliness**	“How often do you feel alone?”	“Very often”, “Often”, “Sometimes”, “Never”	“Very often” or “Often” => often lonely Other = > never or sometimes
**Self-esteem**	Three statements: A) “I like myself “ B) “I am good enough as I am” C) “Peers at my age like me”	“Totally agree”, “Agree”, “Neither agree or disagree”, “Disagree” or “Total disagree”	“Totally agree” or “Agree” to all three questions = > high self-esteem Other = > middle or low
**Self-efficacy**	Two statements: A) “How often can you find a solution to a problem if you try just hard enough?” B) “How often do you reach your goals that you set up?”	“Always”, “Most of the time”, “Sometimes”, “Rarely” or “Never”	“Always” or “Most of the time” to both questions = > high self-efficacy Other = > middle or low
**Social competence**	Three statements: A) “I try to understand my friends when they are sad or angry” B) “I work well with others in a group” C) “I give my opinion when I feel something is unfair”	“Almost never”, “Sometimes”, “Often” or “Almost always”	“Almost always” or “Often” to all three questions = > high social competence. Other = > middle or low

### Participants

**Fig. 1:**
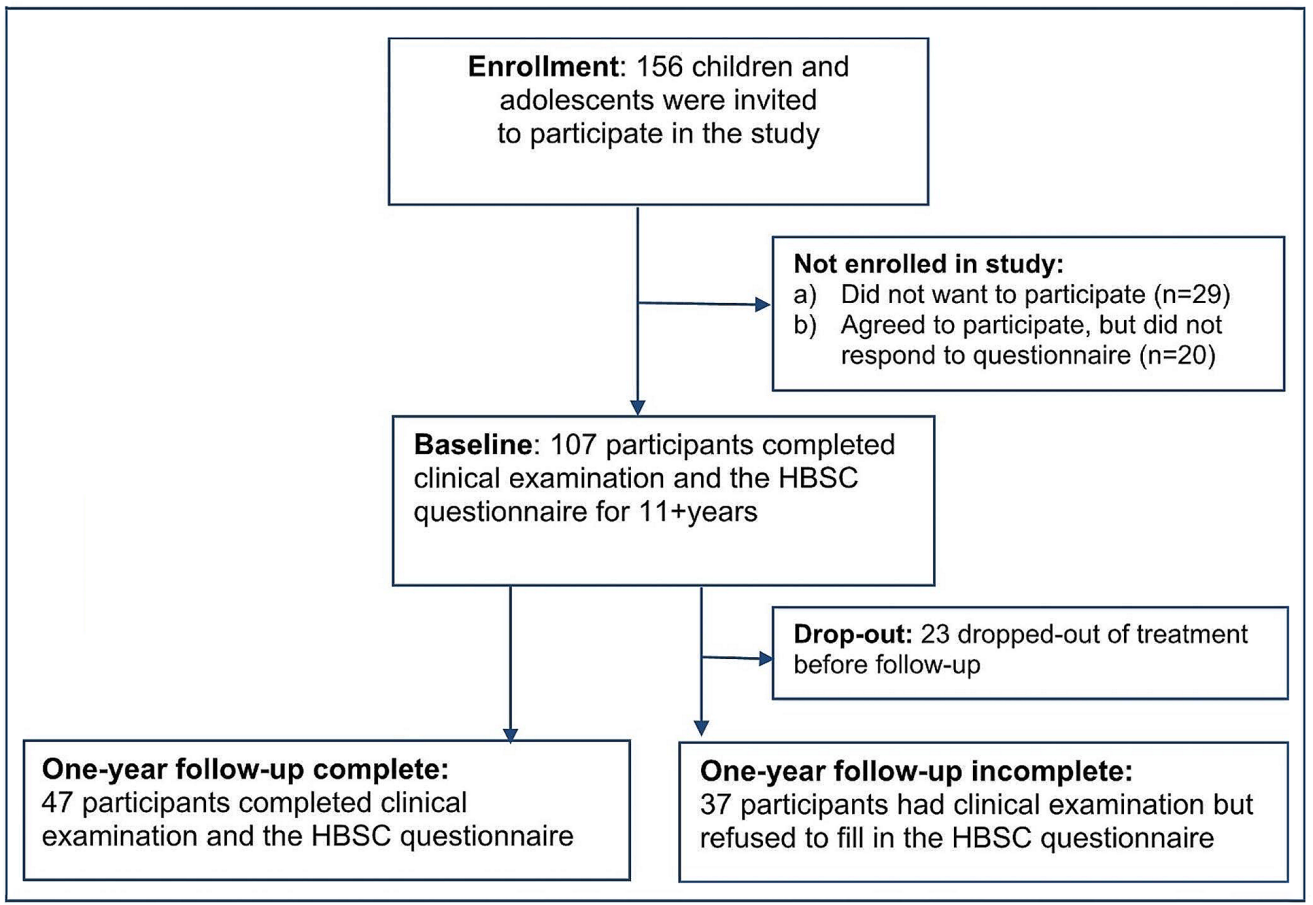
Study overview

During the study period 156 children and adolescents were invited to fill out the HBSC-questionnaire immediate after their first visit in The Children’s Obesity Clinic, and 107 (68,6%) accepted to complete the questionnaire (Fig. 1). Non-participants (*n* = 49) did not want to participate (*n* = 29) or agreed to participate but did not return the questionnaire (*n* = 20) (Fig. 1). Follow-up: Participants who completed the HBSC questionnaire at first visit and after one year follow-up (*n* = 47) were included in follow-up analysis of mental health (Fig. 1).

### Reference population

The reference population was Danish data from the international, cross-sectional Health Behaviour in School-aged Children (HBSC) study [24, 25]. This data collection is conducted every fourth year in each participating country among students aged 11, 13 and 15 years (in Denmark, equivalent to 5th, 7th and 9th grade, respectively) in a random sample of schools. For this study, the 2014 data collection was chosen. Students completed the self-administered, internationally standardized and anonymous HBSC questionnaire at school [26]. The participating schools comprised 5292 students in 248 classes at grade 5, 7 and 9. Of the students present on the day of data collection, 4534 students submitted a satisfactorily completed questionnaire. The response rate was 85.7% (4534/5292) [25].

### Statistics

Clinical data and data from HBSC-questionnaires were entered into a Microsoft Access database and exported to IBM SPSS version 27 for analyses. The data obtained from HBSC questionnaires were integrated with the HBSC dataset from the general population to enable group-comparison analysis.The Pearsons Chi^2^ test and independent samples t-test was used to compare baseline age, BMI-SDS, SES, family structure and presence of psychiatric disease in children who participated and did not participate in the study. The Pearsons Chi^2^ test was further used to compare mental health outcome in children from the Obesity Clinic with the reference population. Logistic regression was performed to test whether gender, age, family structure or BMI-SDS had any effect on mental health in children from the Obesity Clinic. McNemar-test was used to analyse change in mental health from baseline to follow-up. Finally, logistic regression was used to test change in BMI-SDS’s effect on change in mental health from baseline to follow-up.

### Ethics

The study was accepted by Danish Data Protection Authority (j.nr.: 2012-58-0004.). The study did not need acceptance from National Committee on Health Research Ethics (H-2-2014-FSP59å). Written consent was collected from all the participants and their parents. Completing the HBSC questionnaire or not had no consequence for the treatment.

## Results

### Descriptive analysis

The observational study included 107 children and adolescents (54.2% boys) with a mean age 13.2 years and BMI-SDS at 2.9 at baseline. A psychiatric disease was reported by 3.7%, and 67.3% were categorized as having a moderate to high socioeconomic status and 45.8% lived with both parents at baseline. At one year follow-up 47 children and adolescents completed clinical examination and HBSC questionnaire (Fig. 1; Table 2.).

**Table 2:** Characteristics of study population

	Baseline (clinical + HBSC)	Follow-up complete (clinical + HBSC)	Follow-up incomplete (clinical)	Test, *P*-value (complete follow-up vs. incomplete)
**N (%boys)**	107 (♂54.2%)	47 (♂48.9%)	37 (♂62.2%)	
**Age (years)**	13.2 (10.5–17.4)	13.0 (10.5–16.8)	12.9 (10.5–16.5)	*t*(82) = 0.278, *p* = 0.8
**SES**				
**Moderate to high**	72 (67.3%)	32 (68.1%)	26 (70.3%)	X^2^ (1) = 0.46, *p* = 0.8
**Low**	35 (32.7%)	15 (31.9%)	11 (29.7%)	
**Family structure**				
**Lives with both parents**	49 (45.8%)	22 (46.8%)	18 (48.6%)	X^2^ (1) = 0.03, *p* = 0.9
**Disrupted**	58 (54.2%)	25 (53.2%)	19 (51.4%)	
**Psychiatric diseases**	4 (3.7%)	3 (6.4%)	0 (0%)	X^2^ (1) = 2.45, *p* = 0.12
**BMI-SDS (baseline)**	2.9 (1.3-4.)	2.9 (1.8-4.0)	3.0 (1.7-4.0)	*t*(82)=-0.9, *p* = 0.37
**BMI-SDS (follow-up)**		2.6 (1.1-4.0)	2.9 (1.3–4.1)	*t*(82)=-2.2, *p* = 0.03
**Change in BMI-SDS from baseline to follow-up**		-0.3 (-1.5-0.5)	-0.1(-0.7-0.4)	*t*(77.8) = 2.56, *p* = 0.01

### Baseline mental health in children and adolescents with obesity compared to the reference population

Participants from the obesity clinic had significantly increased odds of reporting negative body image (OR 24.3, 95% CI 11.8-50.16), feeling low (OR 1.6, 95% CI 1.0-2.6), and lower odds of reporting a high self-rated health (OR 0.18, 95% CI 0.09–0.35) and a high self-esteem (OR 0.41, 95% CI 0.28–0.61) compared to the reference population.

There was no difference in the two groups with regards to life-satisfaction, social competence, self-efficacy or feeling lonely (all *p* ≥ 0.17) (Table 3).

Supplementary analysis on gender found that both girls and boys from the obesity clinic had significantly increased odds of reporting negative body image (boys: OR 36.7, 95% CI 14.59–92.29, *p <* 0.00, girls: OR 19.12, 95% CI 5.93–61.65, *p <* 0.00) and lower odds of reporting a high self-rated health (boys: OR 0.25, 95% CI 0.12–0.52, *p <* 0.00, girls: OR 0.05, 95% CI 0.007–0.36, *p <* 0.00) and high self-esteem (boys: OR 0.4, 95% CI 0.24–0.68, *p <* 0.00, girls: OR 0.28, 95% CI 0.14–0.56, *p <* 0.00) compared to boys and girls from the reference population. There was no difference when examining gender between the groups with regards to feeling low, life-satisfaction, social competence, self-efficacy or feeling lonely (all *p* ≥ 0.07).

**Table 3:** Baseline mental health in children with obesity compared to HBSC in the reference population

Mental health	Participants *n* = 107	Reference *n* = 4534	Oddsratio (95% CI)	Chi-squared test, *p*-value
**Body image**				
Negative	99 (92.5%)	1519 (33.7%)	24.3 (11.8–50.2)	*p* = 0.00
Normal or positive	8 (7.5%)	2988 (66.3%)		
**Self-rated health**				
High	9 (8.4%)	1555 (34.4%)	0.18 (0.09–0.4)	*p* = 0.00
Middle or low	98 (91.6%)	2960 (65.6%)		
**Life-satisfaction**				
High	34 (31.8%)	1362 (30.1%)	1.08 (0.7–1.6)	*p* = 0.71
Middle or low	73 (68.2%)	3160 (69.9%)		
**Self-esteem**				
High	42 (40.8%)	2461 (62.6%)	0.41 (0.3–0.6)	*p* = 0.00
Middle or low	61 (59.2%)	1469 (37.4%)		
**Social competence**				
High	60 (57.1%)	2184 (54.6%)	1.11 (0.8–1.6)	*p* = 0.6
Middle or low	45 (42.9%)	1817 (45.4%)		
**Self-efficacy**				
High	66 (63.5%)	2893(71%)	0.71 (0.5–1.1)	*p* = 0.09
Middle or low	38 (36.5%)	1180 (29%)		
**Lonely**				
Often	4 (3.8%)	324 (7.2%)	0.51 (0.2–1.4)	*p* = 0.18
Never or sometimes	102 (96.2%)	4182 (92.8%)		
**Feeling low**				
Often	21 (20.2%)	583 (13.5%)	1.62 (1.0-2.6)	*p* = 0.05
Less than every week	83 (79.8%)	3743 (86.5%)		

### Associations between BMI-SDS, age, gender, SES and mental health

A lower BMI-SDS was positively correlated with higher self-efficacy (*p <* 0.04) and higher life satisfaction (*p <* 0.02). BMI-SDS did not have an effect on self-esteem, feeling low, body image, feeling lonely, self-rated health or social competence.

A younger age predicted a higher life satisfaction (*p* < 0.008). Age had no effect on other mental health parameters.

Boys were more likely to rate their self-esteem and self-efficacy as high (*p* < 0.01) and were more likely to feel low (*p* < 0.005) compared to girls. There was no gender difference regarding life-satisfaction, body-image, feeling lonely, self-rated health and social competence.

Living with both parents compared to a disrupted family structure was positively correlated with a higher self-esteem (*p =* 0.01) but had no effect on other mental health parameters (Table S1, see supplementary material).

### Change in mental health from first clinical visit to one year follow-up

There were no significant changes in any of the eight mental health parameters (all *p* ≥ 0.13). (Table 4).

**Table 4:** Change in mental health in participants who completed one-year follow-up

Mental health	Proportion at baseline	Proportion at follow-up	Test of difference between baseline and follow-up proportion. Test, *P*-value
Lonely often (*n* = 47)	0/47 = 0%	4/47 = 8.5%	*p* = 0.13
Negative body image (*n* = 47)	42/47 = 89%	43/47 = 91%	*p* = 1.0
Feeling low often (*n* = 43)	7/43 = 16%	6/43 = 14%	*p* = 1.0
High self-rated health (*n* = 46)	3/46 = 6.5%	9/46 = 19.5%	*p* = 0.11
High self-efficacy (*n* = 45)	30/47 = 66.7%	31/47 = 68.9%	*p* = 1.0
High social competence (*n* = 44)	25/44 = 56.8%	25/44 = 56.8%	*p* = 1.0
High life-satisfaction (*n* = 46)	19/46 = 41%	17/46 = 37%	*p* = 0.79
High self-esteem (*n* = 42)	17/42 = 40%	23/42 = 54.7%	*p* = 0.18

### Change in BMI-SDS’s effect on mental health from baseline to follow-up

An increase in BMI-SDS from baseline to follow-up correlated with significantly lower odds of reporting a high social competence at follow-up (*p* = 0.04, exp 0.095).

There was no effect of change in BMI-SDS scores on the change in the remaining mental health parameters from baseline to follow-up (Table 5.).

**Table 5:** Effect of change in BMI-SDS’s on change in mental health from baseline to follow-up

Mental health parameter	Sig	Exp (B)	95% CI for EXP(B)
Self-rated health	0.08	0.18	0.027–1.214
Life-satisfaction	0.67	0.72	0.15–3.37
Feeling low	0.09	13.79	0.609–312.4
Body image	0.39	2.82	0.27–29.1
Loneliness	0.07	31.2	0.71-1365.7
Self-esteem	0.71	0.74	0.15–3.62
Self-efficacy	0.47	5.28	0.095–2.95
Social competence	0.04	0.095	0.01–0.85

Logistic regression was used to analyze the effect of change in BMI-SDS’s on change in mental health from baseline to follow-up

### Drop out analyses

Baseline sensitivity analyses was performed to compare non-participants (*n* = 29) and non-responders (*n* = 20). Non-responders had higher BMI-SDS (*p* = 0.02) and lower SES-score (*p* = 0.02) compared to non-participants. Therefore, non-responders have not been added to the non-participants group.

The children and adolescents who did not wish to participate (*n* = 29) had a higher presence of psychiatric disease (*p* = 0.04) compared to children and adolescents who participated in the study (*n* = 107), but no difference in age, SES or BMI-SDS. Children and adolescents who participated compared to those who did not respond (*n* = 20) did not differ in any parameters.

Follow up: The HBSC questionnaire was completed by 47 out of 107 participants at follow-up (completers), while non-completers (*n* = 37) did not want to fill in the questionnaire and 23 had dropped out from treatment.

Children and adolescents who had dropped out of the treatment-program before follow-up (*n* = 23) were older (*p* = 0.004) compared to those who completed the program (*n* = 84) (Fig. 1.), but the two groups did not differentiate in baseline BMI-SDS, SES, family structure or psychiatric disease.

Participants who completed the HBSC questionnaire at follow-up compared to those who did not complete the questionnaire at follow-up did not differentiate in age, SES, family structure or presence of psychiatric disease. Baseline BMI-SDS was 2.9 in both groups (see Table 2). At follow-up, those who completed the HBSC questionnaire had lower BMI-SDS compared to the group that did not complete the HBSC questionnaire (BMI-SDS 2.56 versus 2.87, *p* = 0.03).

## Discussion

This study investigated associations between mental health parameters and BMI-SDS in a sample of Danish children and adolescents with obesity compared to a large reference population.

Children and adolescents with obesity had a significantly lower self-rated health, lower self-esteem, negative body image and more often felt low compared to the reference population. These results support previous findings showing that obesity in children is associated with lower self-esteem [27, 28] and lower self-rated health [12].

Actual body weight and body image have shown to be linked [29]. A negative body image may reflect awareness of a weight-issue, but it may also imply that the children have negative emotions concerning their body. Pila et al. (2015) [30] showed that negative body emotions such as body related shame and guilt mediated the relationship between being overweight and having a low self-esteem and suggested it may be beneficial in obesity treatment to reduce negative body-related emotions, in order to gain a positive effect on self-esteem. This is supported by another study [31] showing that improvement in body satisfaction in overweight/obese girls may protect against excessive weight gain.

Self-rated health and physical well-being have in other studies been investigated by using different health-related quality of life questionnaires. Studies using paediatric QOL and KIDSCREEN-27 demonstrated lower physical health related QOL and lower physical well-being in children with obesity compared to children with normal weight [32–34].

Surprisingly, children with obesity and the reference population reported same levels of social competence, life satisfaction and loneliness. These findings seem to contrast with other studies that have found that children and adolescents with obesity report lower QoL, lower psychological well-being and experience more peer relationship problems compared to children with normal weight at same age [34]. While it cannot be excluded that our result might be due to selection bias, we still consider it important since healthcare professionals in the field of paediatric obesity should indeed be focused on children’s strengths and resources. An a priori negative expectation to a child’s well-being and resources might lead to avoidance of starting a conversation about sensitive issues, such as life-satisfaction, friends and relations. The findings are clinically relevant because it highlights the complexity of mental health in children and adolescents with high BMI-SDS and thus the importance of a non-stigmatizing approach in clinical encounters as well as psychosocial support as a mainstay in overweight/obesity treatment.

### Mental health and BMI-SDS

In the study group of children with obesity we found a negative correlation between BMI-SDS and both self-efficacy and life-satisfaction. This is in line with a German study among adolescents (*n* = 1137, 29% with overweight or obesity) who reported a significant association between higher BMI-SDS and lower scores on both physical and psychological well-being [34].

Self-efficacy is one aspect of psychological well-being, describing a person’s conviction that one can successfully execute the behaviour required to reach a specific goal.

The relationship between BMI and self-efficacy in early adolescence has been studied among adolescents, and Steele et al. reported a significant negative relationship between BMI and self-efficacy in early adolescence [13]. Previous studies have included self-efficacy as an outcome for weight loss programs [35, 36]. Roach et al. (2003) showed that using behavioural techniques to improve self-efficacy can be effective in weight loss promotion. They did not observe any greater weight loss, but patients improving their self- efficacy adopted a healthier diet, which is an essential element in long-term weight changes [35]. Our results support that children and adolescents with obesity might benefit from increased behavioural techniques to improve self-efficacy.

### Gender, age and family structure

Among participants from the Obesity Clinic, girls rated their self-esteem and self-efficacy lower and more often felt low compared to boys. Several studies have revealed gender differences in clinical presentation of mental health issues [37], also supported by the Danish HBSC report (2018) [38]. Research on gender influence on mental health in children with obesity is however still sparse.

A lower age was associated with higher life satisfaction among participants from the Obestiy Clinic. This association was also found in the general population in the Danish HBSC study, where BMI was not included in the analysis (2018) [38], suggesting that life satisfaction is higher in younger age groups, irrespective of weight status. However, the German LIFE Child study including 1137 adolescents, 11–18 years of age, found that associations between BMI-SDS and HRQoL were particularly strong in early puberty suggesting that this might be a particularly sensitive period [34].

A disrupted family structure was associated with lower self-esteem. To our knowledge, family structure in relation to obesity and self-esteem has not been thoroughly investigated. It has been reported that children experiencing a family disruption have an increased risk of obesity in two years leading up to the disruption as well as after the disruption [39, 40]. Further studies are needed to investigate the effect of family structure and gender on child obesity and self-esteem.

### Change in mental health during one-year treatment

We did not find any significant change in mental health over a year, but a tendency to a higher proportion reporting high self-rated health after one year. Previous reviews investigating mental health parameters in children’s obesity treatment programs have found inconsistent results. Gow et al. (2020) found improvement in self-esteem and body-image over time [41], while Murray et al. [42] did not find any change in self-esteem. The divergent findings may be due to small study population and high heterogeneity in studies.

### Drop-out

One year after study start, 84 (78%) of the participants were followed up with a clinical examination, but only 47 (55%) completed the HBSC questionnaire. The high proportion of children who refused to complete the questionnaire at follow-up might be explained by their previous experience with the comprehensive questionnaire, which seemed mentally exhausting some participants.

Previous studies have also reported high dropout rates in children’s obesity treatment programs ranging from 4 to 83% [43]. A systematic review found higher dropout rates to be associated with ethnicity and low compliance associated with low SES in paediatric weight management programs. This study did not find any associations between dropout and age, SES, family structure or psychiatric disease, but found an increased weight-loss in participants compared to children who dropped out.

### Strengths and limitations

In this study, mental health in children and adolescents was evaluated using the HBSC questionnaire. To our knowledge it is the first time this method has been used specifically in children and adolescents with obesity. A great strength using the HBSC questionnaire is the validated method and the large reference data. The questionnaire was completed in a standardized, calm setup in the obesity clinic after the clinical consultation. A limitation to this setup was the observation that some study participants seemed inattentive during completion of the questionnaire, possibly explained by a demanding process of participating in the clinical encounter in the obesity clinic. This may have affected their ability to fill out a comprehensive questionnaire.

## Conclusion

Children and adolescents with obesity are at increased risk of lower self-esteem, lower self-rated health, more often feeling low and having a negative body image compared to their peers. The study did not find any change in mental health parameters after one-year treatment in a chronic care program. These results suggest that mental health care needs to be addressed clearly in future treatment protocols of children and adolescents with obesity.

At the same time, the study also showed no differences between the two groups in social competence, feeling lonely or life-satisfaction. This indicates that many children with obesity have psychosocial resources matching their peers. Overlooking these resources may lead to stigmatization and unused potentials in supporting a healthier lifestyle. Future studies are recommended exploring these resources in depth and exploring ways to utilize them in clinical settings.

### Electronic supplementary material

Below is the link to the electronic supplementary material.

Supplementary Material 1

## Data Availability

Data and materials can be requested from corresponding author, Katrine Decker Iversen.

## Abbreviations

HBSC: Health Behaviour in School-aged Children
BMI-SDS: Body Mass Index Standard Deviation Score
SES: Socio Economic Status
