# Supplementary material for: Mental health and BMI in children and adolescents during one year in obesity treatment
Source: BMC Pediatr. 2024 Jun 26;24:406. doi: 10.1186/s12887-024-04835-7 (PMC11201871; doi:10.1186/s12887-024-04835-7)
Supplement: Supplementary file 1 — Supplementary Material 1 [file 12887_2024_4835_MOESM1_ESM.docx]

Supplementary material

Table S1

*Table S1, supplementary: Associations between BMI-SDS, age, gender, family structure and mental health parameters in children and adolescents with obesity*

| Logistic regression predicting mental health based on gender, BMI-SDS, age and family structure | | | | |
| --- | --- | --- | --- | --- |
| Mental health | Predictor | Sig | Exp(B) | 95% CI for EXP(B) |
| Self-esteem | Gender | 0.001 | 0.232 | 0.097-0.556 |
|  | BMI-sds | 0.104 | 0.528 | 0.245-1.141 |
|  | Age | 0.067 | 0.798 | 0.627-1.015 |
|  | Family structure | 0.011 | 0.347 | 0.154-0.783 |
| Self-efficacy | Gender | 0.05 | 0.443 | 0.197-1.001 |
|  | BMI-sds | 0.043 | 0.442 | 0.2-0.976 |
|  | Age | 0.912 | 1.013 | 0.805-1.275 |
|  | Family structure | 0.944 | 0.971 | 0.435-2.167 |
| Feeling low | Gender | 0.012 | 3.788 | 1.334-10.758 |
|  | BMI-sds | 0.432 | 1.420 | 0.593-3.401 |
|  | Age | 0.862 | 1.025 | 0.778-1.350 |
|  | Family structure | 0.264 | 1.773 | 0.649-4.840 |
| Life-satisfaction | Gender | 0.513 | 0.760 | 0.334-1.731 |
|  | BMI-sds | 0.019 | 0.382 | 0.171-0.851 |
|  | Age | 0.005 | 0.670 | 0.505-0.889 |
|  | Family structure | 0.312 | 0.656 | 0.290-1.486 |
| Body image | Gender | 0.626 | 1.447 | 0.328-6385 |
|  | BMI-sds | 0.708 | 0.778 | 0.209-2.897 |
|  | Age | 0.255 | 0.793 | 0.531-1.183 |
|  | Family structure | 0.105 | 3.907 | 0.751-20.231 |
| Feeling lonely | Gender | 0.997 | 1468 | 0.00-1 |
|  | BMI-sds | 0.084 | 6.297 | 0.783.50.652 |
|  | Age | 0.183 | 1.462 | 0.836-2.557 |
|  | Family structure | 0.403 | 2.667 | 0.268-26.5 |
| Self-rated health | Gender | 0.059 | 0.13 | 0.016-1.081 |
|  | BMI-sds | 0.610 | 1.384 | 0.397-4.829 |
|  | Age | 0.176 | 0.718 | 0.445-1.160 |
|  | Family structure | 0.202 | 0.391 | 0.092-1.654 |
| Social competence | Gender | 0.396 | 1.403 | 0.642-3.068 |
|  | BMI-sds | 0.701 | 0.866 | 0.415-1.806 |
|  | Age | 0.302 | 1.127 | 0.898-.1415 |
|  | Family structure | 0.572 | 1.251 | 0.576-2.718 |
